# Efficacy and Safety of Hypomethylating Agents in Chronic Myelomonocytic Leukemia: A Single-Arm Meta-analysis

**DOI:** 10.1055/s-0042-1744157

**Published:** 2022-04-08

**Authors:** Xinhui Zheng, Liwei Lv, Xiangjun Li, Erlie Jiang

**Affiliations:** 1 State Key Laboratory of Experimental Hematology, National Clinical Research Center for Blood Diseases, Haihe Laboratory of Cell Ecosystem, Institute of Hematology and Blood Diseases Hospital, Chinese Academy of Medical Sciences and Peking Union Medical College, Tianjin, China; 2 Department of Hematology, Beijing Tiantan Hospital, Capital Medical University, Beijing, China; 3 Department of Breast Surgery, Affiliated Hospital of Qingdao University, Qingdao University, Qingdao, China

**Keywords:** chronic myelomonocytic leukemia, hypomethylating agents, meta-analysis, myelodysplastic syndromes, single-arm, azacitidine, decitabine

## Abstract

**Background** Chronic myelomonocytic leukemia (CMML) is a myeloid neoplasm with features of the myelodysplastic syndromes (MDSs) and myeloproliferative neoplasm presenting with peripheral blood monocytosis and an inherent risk for transformation to acute myeloid leukemia, while the abnormal DNA methylation plays a critical role in the pathogenesis of MDS, which is a disease of disordered differentiation. Recently, with the rapid development of molecular biology, hypomethylating agents (HMAs) for the treatment of MDS has gradually become a research focus. The objective of this study was to evaluate the benefits and risks of HMAs for patients with CMML.

**Materials and Methods** PubMed, Embase, the Cochrane Library, and three Chinese databases were searched for studies published before November 2020 that used HMAs in CMML.

**Results** The pooled objective response rate (ORR), complete response (CR), and partial response (PR) were 50.0, 21.0, and 2.0%, respectively. The proportion of patients with minor response (MR) was significantly higher for decitabine (DAC) than for azacitidine (AZA). There was no significant difference in hematologic improvement, ORR, CR, and PR rates between the DAC and AZA groups. Hematological toxicity included neutropenia grade 3/4 (14.0%), anemia grade 3/4 (17.0%), and thrombocytopenia grade 3/4 (22.0%).

**Conclusion** This study showed that HMAs were effective and safe in the treatment of CMML, but large multicenter study would be needed to confirm the efficacy of HMAs for the treatment of CMML with different risk level and genetic abnormality, to support individualization treatment theoretically.

## Introduction

Chronic myelomonocytic leukemia (CMML) is a myeloid neoplasm with features of the myelodysplastic syndromes (MDSs) and myeloproliferative neoplasm presenting with peripheral blood (PB) monocytosis and an inherent risk for transformation to acute myeloid leukemia (AML). 1 In the 2016 World Health Organization (WHO) classification of myeloid neoplasms, CMML is defined as a clonal hematopoietic stem cell disorder characterized by the presence of sustained (more than 3 months) PB monocytosis along with dysplastic features in the bone marrow (BM). 2 And based on blast cells percent in PB and BM, CMML can be divided into CMML-0, CMML-1, and CMML-2. 2

In the late 1990s, chemotherapy such as etoposide, cytarabine, and all-trans retinoic acid is the major treatment options. Unfortunately, response rates in these trials were disappointing. At this time, allogeneic hematopoietic stem cell transplantation (allo-HSCT) remained the only curative therapeutic modality for patients with CMML. However, it was not the first choice for clinical treatment because it was costly and challenging to find a suitable donor, and many patients were not eligible for allo-HSCT. 3 4 5

The abnormal DNA methylation plays a critical role in the pathogenesis of MDS, which is a disease of disordered differentiation. Recently, with the rapid development of molecular biology, hypomethylating agents (HMAs) for the treatment of MDS has gradually become a research focus. 6 7 In fact, the U.S. Food and Drug Administration (FDA) has approved two representative HMAs, azacitidine (AZA) and decitabine (DAC), for treating MDS. Surprisingly, several previous clinical trials have shown that HMAs demonstrated improved outcomes for patients with CMML when compared with conventional care regimens.

Since the FDA approved two HMAs, AZA and DAC, for treatment of patients with MDS, HMAs have been widely used for the management of patients with CMML. 8 9 However, only a few patients with CMML were included in the studies for the use of HMAs in MDS and response rates for CMML were not reported separately. Only small-sized studies have shown the evidence for the effectiveness of HMAs in CMML. There were differences in the response rates and overall survival in different studies. Some researches indicated there were differences in efficacy between CMML-1 and CMML-2, AZA and DAC, or different mutations. However, there is no definitive conclusion. 10 Defining the toxicities, response rates and survival rates of HMA and influencing factors of efficacy can help guide clinicians to individualized medication and improve the survival rate and quality of life of CMML patients.

Therefore, this study aimed at evaluating the impact of hypomethylation therapy for the treatment of patients with CMML, focusing on long-term outcomes of patients by comprehensively collecting clinical studies on hypomethylation therapy in such clinical settings.

## Materials and Methods

### Search Strategy

Three investigators performed a comprehensive search using several databases before November 2020: MEDLINE, PubMed, Embase, Cochrane Center Register of Controlled Trials, Wanfang Data Knowledge Service Platform, China National Knowledge Infrastructure, and Chinese BioMedical Literature Database. Eligible studies were relevant clinical trials on patients with CMML treated with HMAs. The search keywords were “chronic myelomonocytic leukemia” and “hypomethylating agents,” and the search strategy in PubMed is shown in Supplementary Table S1 , available in online version only.

### Inclusion and Exclusion Criteria

Studies were eligible if they met the following criteria in this meta-analysis: (1) patients aged ≥18 years with a diagnosis of CMML according to WHO criteria; (2) untreated or previously treated with hydroxyurea or etoposide given orally; and (3) nonintensive chemotherapy or intensive chemotherapy given more than 1 month and to have recovered from the side effects of prior therapy before inclusion. While the exclusion criteria were as follows: (1) patients with a myeloproliferative or MDS other than CMML; (2) acute blastic transformation of CMML with BM blast cells >20%; and (3) allogenic stem cell transplant with an identified donor, intensive chemotherapy in the last 1 month and previous treatment with a HMA.

### Quality Assessment

The methodological index for nonrandomized studies (MINORS) was used to assess the quality of nonrandomized studies (single-arm studies). 11 An adjusted version of the Joanna Briggs Institute (JBI) critical appraisal checklist for case series was used to assess the quality of the single-arm open-label studies.

### Data Extraction

The following data were extracted from each study: the first author's name, year of publication, phase of trials, median age, treatment and dosing regimens, median treatment duration, number of patients available for analysis, and main outcomes. Main outcomes were the rates of hematologic response (objective response rate [ORR]; complete response [CR]; partial response [PR]) according to the International Working Group 2006 criteria. 12 The additional outcomes were hematologic improvement (HI); minor regression (MR); disease progression (PD) rate, grade 3/4 neutrophil toxicity rate, grade 3/4 anemia toxicity rate, grade 3/4 platelet toxicity rate, infection rate, rate of bleeding events, and rate of cardiovascular events.

### Statistical Analysis

For data collection, we used Microsoft Excel and all statistical analysis was performed using STATA12. Three independent investigators extracted data from each eligible study using a standardized data extraction form. To calculate the pooled rates of the ORR, CR, PR, MR, HI, PD, rate of grade 3/4 neutrophil toxicity, rate of grade 3/4 anemia toxicity, rate of grade 3/4 platelet toxicity, infection rate, rate of bleeding events, and rate of cardiovascular events, a random-effect model according to the DerSimonian–Laird approach with the Freeman–Tukey double arcsine transformation with 95% confidence interval (CI) was used. Because of the significant heterogeneity expected among the participants of all the included studies, we used the random/effect model, rather than the fixed/effect model. Cochran's Q test and *I* ^2^ statistic were applied to determine the between-study heterogeneity. A guide to the interpretation of *I* ^2^ values was as follows: *I* ^2^ ≥ 25%, low heterogeneity; *I* ^2^ ≥ 50% moderate heterogeneity; and *I* ^2^ ≥ 75%, high heterogeneity. Moreover, Begg's and Egger's tests were applied to assess the potential publication bias.

## Results

### Study Selection and Characteristics

**Fig. 1:**
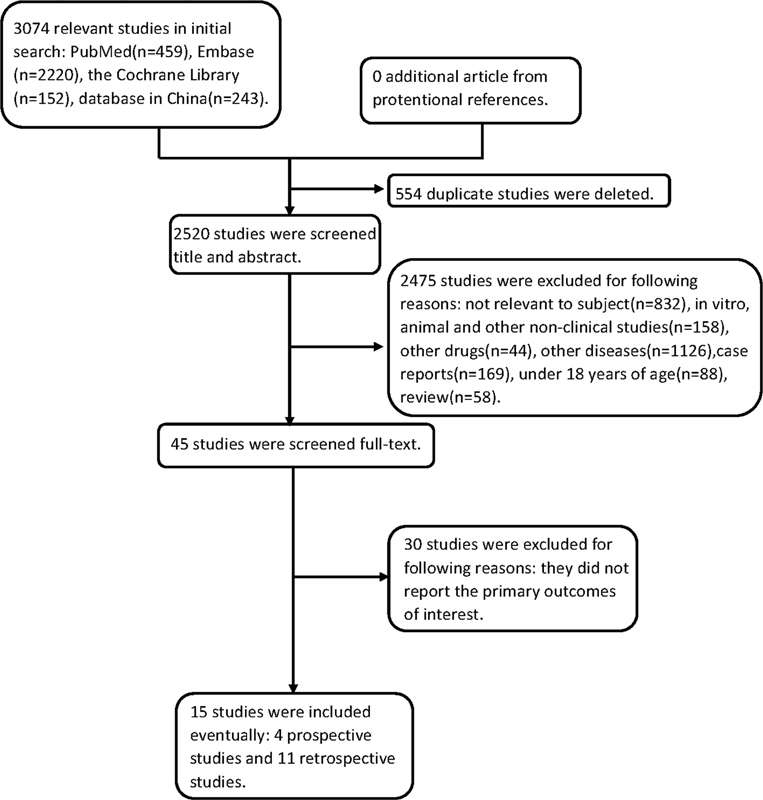
Study selection process.

In the initial search, 3,074 relevant articles were identified (2,831 English articles and 243 Chinese articles). After the exclusion of 554 duplicated articles using EndNote X9 software, two independent reviewers performed a title and abstract screening on the 2,520 residual articles. A total of 2,475 studies were excluded for following reasons: not relevant to subject ( *n* = 832), in vitro, animal, and other nonclinical studies ( *n* = 158), other drugs ( *n* = 44), other diseases ( *n* = 1,126), case reports ( *n* = 169), under 18 years of age ( *n* = 88), and review ( *n* = 58). The remaining articles were then reviewed in full text and 30 of them were excluded because they did not report the primary outcomes of interest. The 15 remaining studies satisfied the eligibility criteria and were included in the meta-analysis, including 4 prospective cohort studies 13 14 15 16 and 11 retrospective studies. 17 18 19 20 21 22 23 24 25 26 27 Fig. 1 demonstrates the literature review and identification process. A total of 647 patients with CMML across 15 cohort studies were planned to be included in this meta-analysis to evaluate the efficacy and toxicity of the HMAs regimens. Patient's age ranged from 18 to 89 years, with a male predominance (68.01%). Baseline clinical characteristics of these patients are summarized in Supplementary Table S1 .

### Quality Assessment

The MINORS index score was used to assess four single-arm studies 13 14 15 16 from 12 to 13 points, which were suitable for the present meta-analysis ( Supplementary Table S2, A ). Eleven retrospective studies 17 18 19 20 21 22 23 24 25 26 27 without comparison were included after they were tested based on the JBI critical appraisal checklist for case series ( Supplementary Table S2, B ).

### Efficacy

#### Tumor Response

**Fig. 2:**
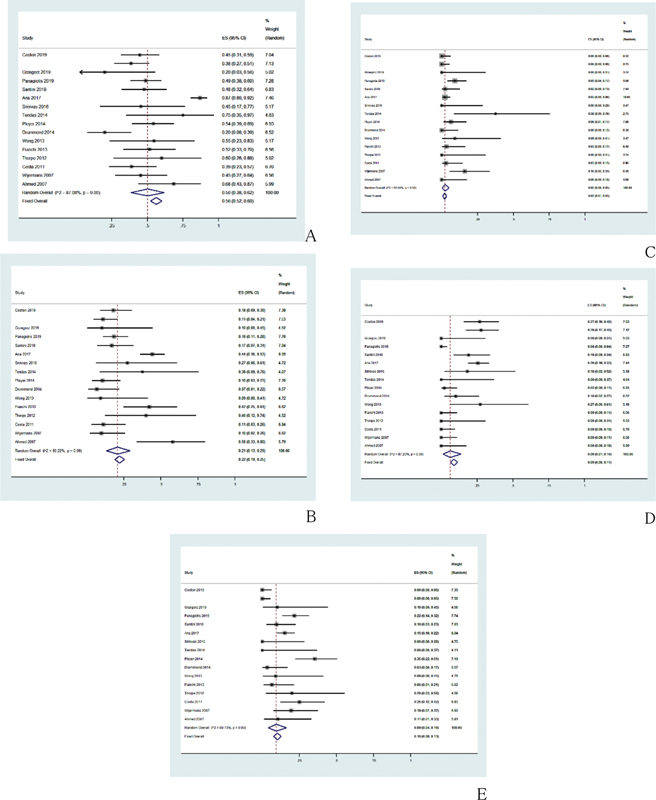
Pooled results of response in total. ( **A** ) Pooled results of ORR in total was 50% (95% CI, 38–62%, *I* ^2^ = 87.08%). ( **B** ) The pooled CR rate after treatment with HMAs regimen was 21% (95% CI, 13–29%, *I* ^2^ = 80.22%). ( **C** ) Pooled results of PR in total was 2% (95% CI, 0–5%, *I* ^2^ = 55.04%). ( **D** ) Pooled results of MR in total was 6% (95% CI, 1–14%, *I* ^2^ = 87.20%). (E) Pooled results of HI in total was 9% (95% CI, 4–16%, *I* ^2^ = 80.73%). CI, confidence interval; CR, complete response; HI, hematologic improvement; HMAs, hypomethylating agents; ORR, objective response rate; MR, minor response; PR, partial response.

The pooled CR rate after treatment with HMAs regimen was 21% (95% CI, 13–29%, *I* ^2^ = 80.22%; Fig. 2B ), while the pooled ORR was 50% (95% CI, 38–62%, *I* ^2^ = 87.08%; Fig. 2A ). The PR rate was 2% (95% CI, 0–5%, *I* ^2^ = 55.04%; Fig. 2C ). The MR rate was 6% (95% CI, 1–14%, *I* ^2^ = 87.20%; Fig. 2D ). The HI rate was 9% (95% CI, 4–16%, *I* ^2^ = 80.73%; Fig. 2E ).

#### Subgroup Analysis

**Fig. 3:**
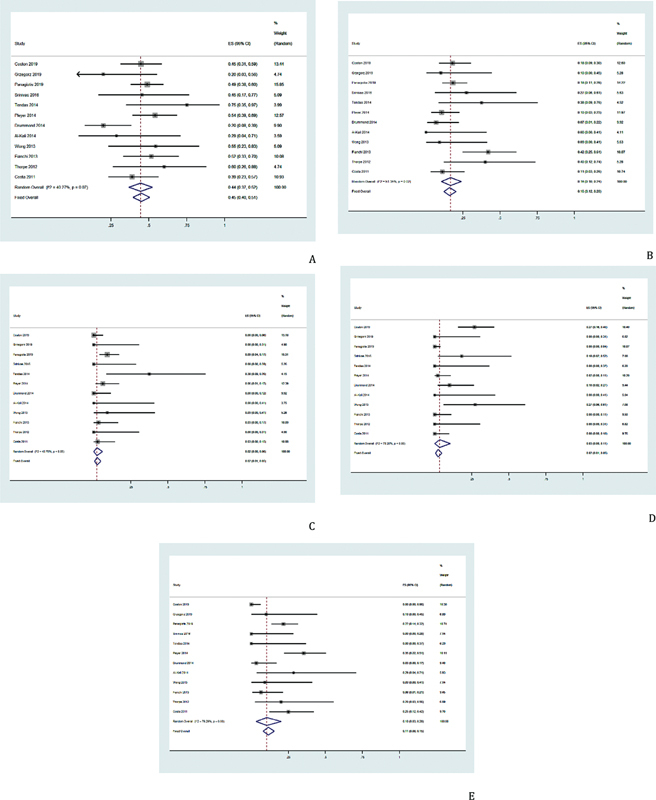
Pooled results of tumor response in the AZA group. ( **A** ) Pooled results of ORR in the AZA group was 45% (95% CI, 40–51%, *I* ^2^ = 40.27%). ( **B** ) The pooled CR rate after treatment with the AZA agents was 16% (95% CI, 10–24%, *I* ^2^ = 51.35%). ( **C** ) Pooled results of PR in the AZA group was 2% (95% CI, 1–5%, *I* ^2^ = 43.75%). ( **D** ) Pooled results of MR in the AZA group was 3% (95% CI, 0–11%, *I* ^2^ = 78.20%). ( **E** ) Pooled results of HI in the AZA group was 10% (95% CI, 3–20%, *I* ^2^ = 79.29%). AZA, azacitidine; CI, confidence interval; CR, complete response; HI, hematologic improvement; ORR, objective response rate; MR, minor response; PR, partial response.

AZA therapy was given in 10 studies, 16 18 19 20 21 22 23 24 25 26 and the ORR, CR, PR, MR, and HI were 45% (95% CI, 40–51%, *I* ^2^ = 40.27%; Fig. 3A ), 16% (95% CI, 10–24%, *I* ^2^ = 51.35%; Fig. 3B ), 2% (95% CI, 1–5%, *I* ^2^ = 43.75%; Fig. 3C ), 3% (95% CI, 0–11%, *I* ^2^ = 78.20%; Fig. 3D ), and 10% (95% CI, 3–20%, *I* ^2^ = 79.29%; Fig. 3E ), respectively.

**Fig. 4:**
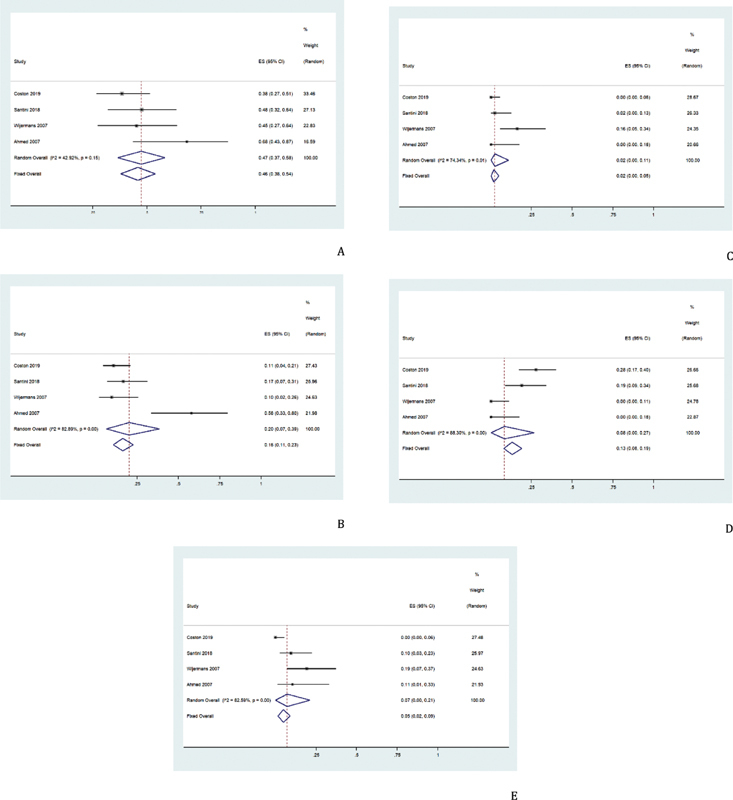
Pooled results of tumor response in the DAC group. ( **A** ) Pooled results of ORR in the DAC group was 46% (95% CI, 38–54%, *I* ^2^ = 42.92%). ( **B** ) The pooled CR rate after treatment with the DAC agents was 20% (95% CI, 7–39%, *I* ^2^ = 82.89%). ( **C** ) Pooled results of PR in the DAC group was 2% (95% CI, 0–11%, *I* ^2^ = 74.34%). ( **D** ) Pooled results of MR in the DAC group was 8% (95% CI, 0–27%, *I* ^2^ = 88.30%). ( **E** ) Pooled results of HI in the DAC group was 7% (95% CI, 0–21%, *I* ^2^ = 82.59%). CI, confidence interval; CR, complete response; DAC, decitabine; HI, hematologic improvement; ORR, objective response rate; MR, minor response; PR, partial response.

DAC therapy was given in four studies, 13 15 18 27 28 and the ORR, CR, PR, MR, and HI were 46% (95% CI, 38–54%, *I* ^2^ = 42.92%; Fig. 4A ), 20% (95% CI, 7–39%, *I* ^2^ = 82.89%; Fig. 4B ), 2% (95% CI, 0–11%, *I* ^2^ = 74.34%; Fig. 4C ), 8% (95% CI, 0–27%, *I* ^2^ = 88.30%; Fig. 4D ), and 7% (95% CI, 0–21%, *I* ^2^ = 82.59%; Fig. 4E ), respectively.

**Fig. 5:**
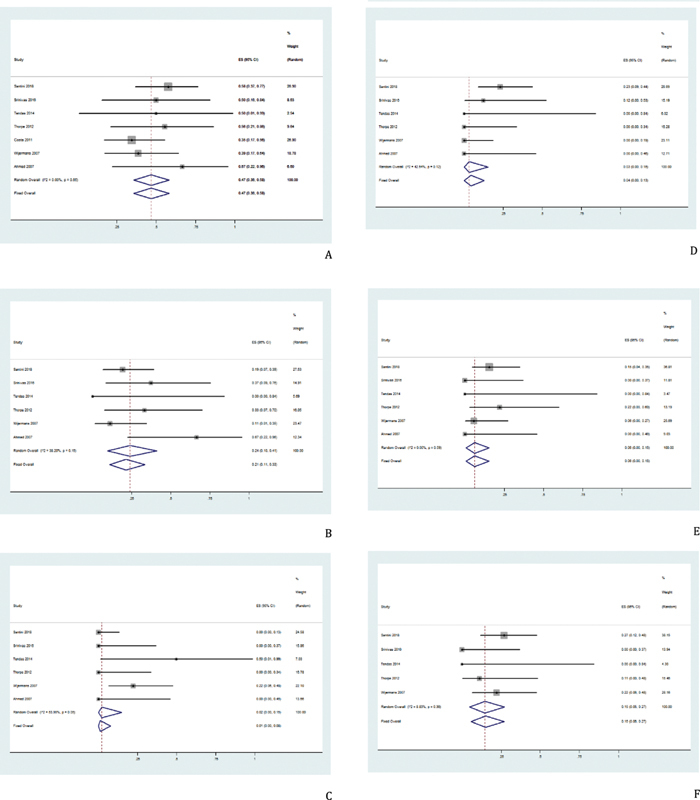
Pooled results of tumor response in the CMML-1 group. ( **A** ) Pooled results of ORR in the CMML-1 group was 47.0% (95% CI, 36.0–58.0%, *I* ^2^ = 0.00%). ( **B** ) The pooled CR rate in the CMML-1 group was 21.0% (95% CI, 11.0–33.0%, *I* ^2^ = 38.20%). ( **C** ) Pooled results of PR in the CMML-1 group was 2.0% (95% CI, 0.0–15.0%, *I* ^2^ = 53.99%). ( **D** ) Pooled results of MR in the CMML-1 group was 4.0% (95% CI, 0.0–13.0%, *I* ^2^ = 42.54%). ( **E** ) Pooled results of HI in the CMML-1 group was 6.0% (95% CI, 0.0–15.0%, *I* ^2^ = 0.00%). ( **F** ) Pooled results of PD in the CMML-1 group was 15.0% (95% CI, 6.0–27.0%, *I* ^2^ = 8.83%). CI, confidence interval; CMML, chronic myelomonocytic leukemia; CR, complete response; HI, hematologic improvement; ORR, objective response rate; MR, minor response; PD, disease progression; PR, partial response.

Eight studies 13 15 16 21 22 25 26 27 reported the outcomes of patients with CMML-1 and CMML-2, respectively. In CMML-1 group, the rate of ORR was 47.0% (95% CI, 36.0–58.0%, *I* ^2^ = 0.00%; Fig. 5A ), the rate of CR was 21.0% (95% CI, 11.0–33.0%, *I* ^2^ = 38.20%; Fig. 5B ), the rate of PR was 2.0% (95% CI, 0.0–15.0%, *I* ^2^ = 53.99%; Fig. 5C ), the rate of MR was 4.0% (95% CI, 0.0–13.0%, *I* ^2^ = 42.54%; Fig. 5D ), the rate of HI was 6.0% (95% CI, 0.0–15.0%, *I* ^2^ = 0.00%; Fig. 5E ), and the rate of PD was 15.0% (95% CI, 6.0–27.0%, *I* ^2^ = 8.83%; Fig. 5F ). In CMML-2 group, the rate of ORR was 54.0% (95% CI, 37.0–69.0%, *I* ^2^ = 18.61%; Supplementary Fig. S1A , available in online version only), the rate of CR was 27.0% (95% CI, 3.0–61.0%, *I* ^2^ = 65.90%; Supplementary Fig. S1B , available in online version only), the rate of PR was 3.0% (95% CI, 0.0–15.0%, *I* ^2^ = 0.00%; Supplementary Fig. S1C , available in online version only), the rate of MR was 1.0% (95% CI, 0.0–10.0%, *I* ^2^ = 0.00%; Supplementary Fig. S1D , available in online version only), the rate of HI was 1.0% (95% CI, 0.0–20.0%, *I* ^2^ = 51.07%; Supplementary Fig. S1E , available in online version only), and the rate of PD was 13.0% (95% CI, 1.0–30.0%, *I* ^2^ = 34.58%; Supplementary Fig. S1F , available in online version only). There was no significant difference in ORR and PD rates between the CMML-1 and CMML-2 groups ( Supplementary Fig. S2 , available in online version only).

### Safety

Seven studies 13 14 16 20 21 23 25 reported the hematological toxicity. The pooled rate of grade 3/4 neutrophil toxicity was 14.0% (95% CI, 3.0–30.0%, *I* ^2^ = 83.51%; Supplementary Fig. S3A , available in online version only). The rate of grade 3/4 anemia toxicity was 17.0% (95% CI, 6.0–31.0%, *I* ^2^ = 75.59%; Supplementary Fig. S3B ). The rate of grade 3/4 platelet toxicity was 22.0% (95% CI, 8.0–40.0%, *I* ^2^ = 81.73%; Supplementary Fig. S3C ).

Five studies 13 17 20 21 27 reported the nonhematological toxicity. The pooled rate of infection was 11% (95% CI, 5–18%, *I* ^2^ = 0.00%; Supplementary Fig. S3D , available in online version only). The pooled rate of bleeding was 3% (95% CI, 0–10%, *I* ^2^ = 0.00%; Supplementary Fig. S3E , available in online version only). The pooled rate of cardiovascular diseases was 9% (95% CI, 4–16%, *I* ^2^ = 0.00%; Supplementary Fig. S3F , available in online version only).

### Publication Bias

The publication bias was assessed using Egger's publication bias plot and Begg's funnel plot. Significant publication bias was not shown in the pooled OR among included studies, with *p* = 0.114 for Egger's test and *p* = 0.416 for Begg's test. Similarly, publication bias was not significant in CR with Egger's test ( *p* = 0.513) and Begg's test ( *p* = 0.278), PR with Egger's test ( *p* = 0.385) and Begg's test ( *p* = 0.241), MR with Egger's test ( *p* = 0.134) and Begg's test ( *p* = 0.928), and HI with Egger's test ( *p* = 0.492) and Begg's test ( *p* = 0.718).

## Discussion

HMAs have been widely used in clinical practice, since they were approved for use in CMML. However, early significant studies that confirmed the efficacy and safety of these agents in MDS had included only a small number of patients with CMML, and there were just a few specific reports on CMML patients. 29 30

In recent years, the CR greatly ranged from 25 to 70% in different researches about the efficacy and safety of HMA in CMML. This study systematically evaluated the efficacy and safety of the HMA treatment for patients with CMML.

This meta-analysis included a total of 15 studies, involving 647 patients. The ORR in the AZA group was 46.0%, similar with the ORR (41.2–45.8%) of patients with MDS, AML, CMML in a meta-analysis reported by Shapiro and Lazo-Langner. 31 But the rate was significantly lower than the ORR (73%) of AZA treatment for patients with MDS, which indicated that the efficacy of AZA for CMML was far away from that for MDS. The ORR in the DAC groups was 44.0%, similar to the ORR (51%) of patients with MDS in a meta-analysis reported by Yang et al dosages of DAC in treating MDS: a meta-analysis. 32 And the CR rates of AZA and DAC were both low, ∼20%.

Our results demonstrated that HMA had certain curative effect, but there were also some limitations. Therefore, to improve outcome, it was very important to seek for more safe and effective prevention and treatment. AZA and DAC were considered completely similar drugs; however, AZA and DAC achieved their active forms through different metabolic pathways. 33 And in vitro experiments on leukemia cells had shown that AZA and DAC had significantly different effects on cell viability, protein synthesis, cell cycle, and gene expression. 34 However, no matter whether it was MDS or CMML, it had not been determined which of AZA or DAC was better. In this study, the rate of ORR of AZA and DAC was similar. There was still a lack of clinical controlled trials of two demethylation drugs to compare the difference in efficacy between the two.

In subgroup analysis between patients with CMML-1 and CMML-2, the ORR and CR rate of CMML-2 were higher than CMML-1, while the PD rate of CMML-2 was lower than CMML. But the difference was not statistically significant. The results showed that the HMAS was effective to a certain extent in the treatment of CMML-1 and CMML-2; however, due to relatively few studies, it was unclear whether there was a difference in efficacy between CMML-1 and CMML-2. Simultaneously, the efficacy for the patients with different genetic mutations was still unknown because there were few relevant researches.

Our meta-analysis of adverse reactions showed that the incidence of neutropenia, anemia, and thrombocytopenia which combined to grade 3/4 hematological adverse reactions were 14.0, 17.0, and 22.0%, respectively. The incidence were lower than the rates in the treatment for MDS and AML, which were 45.0, 27, and 38%, respectively. 35 Serious nonhematological adverse events are rare, mainly included infection and bleeding, corelated with drug-induced suppression of the BM, suggesting that attention should be paid to the prevention of infection and bleeding in clinical medication, timely blood monitoring, and supportive treatment.

This study had some limitations. Most of the included studies were retrospective analysis, and as single-arm trials lacked control groups, it was impossible to correctly assess the difference in efficacy between the HMA agents and other treatment methods. In addition, there were few comparative studies on the efficacy of HMAs on CMML with different subtypes and different gene mutations, so it was impossible to conduct more in-depth subgroup analysis.

## Conclusion

This study showed that the HMASs are effective and safe in the treatment of CMML. Large multicenter study will be needed to confirm the efficacy of HMAs for the treatment CMML with different risk level and genetic abnormality, to support individualization treatment theoretically. At the same time, many studies indicate that the efficacy of HMAs for the treatment of CMML is limited, so it is still the key point to find new treatment plan.
