# Supplementary material for: Efficacy and Safety of Hypomethylating Agents in Chronic Myelomonocytic Leukemia: A Single-Arm Meta-analysis
Source: Glob Med Genet. 2022 Apr 8;9(2):141–51. doi: 10.1055/s-0042-1744157 (PMC9192189; doi:10.1055/s-0042-1744157)
Supplement: Supplementary file 1 — Supplementary Material [file 10-1055-s-0042-1744157-s2100069.pdf]

Supplementary Material

Search (((((((((((((((((((((((((“myeloproliferative diseases”[-Title/Abstract]) OR “myeloproliferative neoplasm”[Title/Abstract]) OR “myeloproliferative disorder”[Title/Abstract]) OR “myeloproliferative disorders”[Title/Abstract]) OR “chronic myelomonocytic leukemia”[Title/Abstract]) OR “chronic myelomonocytic leukaemia”[Title/Abstract]) OR CMML[Title/Abstract]) OR CMMoL[Title/Abstract]) OR “myelomonocytic leukemia”[Title/Abstract]) OR “myelomonocytic leukaemia”[Title/Abstract]) OR aCML[Title/Abstract]) OR “atypical chronic myeloid leukemia”[Title/Abstract]) OR “atypical chronic myeloid leukaemia”[Title/Abstract]) OR “atypical chronic myelogenous leukaemia”[Title/Abstract]) OR “atypical chronic myelogenous leukemia”[Title/Abstract]) OR “chronic myeloid leukemia”[Title/Abstract]) OR “chronic myeloid leukaemia”[Title/Abstract]) OR “neutrophilic leukemia”[Title/Abstract]) OR “neutrophilic leukaemia”[Title/Abstract]) OR “Chronic Neutrophilic Leukemia”[Title/Abstract])) OR “Leukemia, Neutrophilic, Chronic”[Mesh]) OR “Leukemia, Myelomonocytic, Chronic”[-Mesh]) OR “Myeloproliferative Disorders”[Mesh]) OR “Myelodysplastic-Myeloproliferative Diseases”[Mesh])) AND (((((((((((((((((((((((((((((((“Hypomethylating agent”[Title/Abstract]) OR “Hypomethylating agents”[Title/Abstract]) OR “Hypomethylating drug”[Title/Abstract]) OR “Hypomethylating drugs”[Title/Abstract]) OR “Hypomethylating medication”[Title/Abstract]) OR HMA[Title/Abstract]) OR Azacitidine[Title/Abstract]) OR “2 deoxy 5 azacytidine”[Title/Abstract]) OR “5 azadeoxycytidine”[Title/Abstract]) OR “5 azadesoxycytidine”[Title/Abstract]) OR Azacitidin[Title/Abstract]) OR azacyd[Title/Abstract]) OR Azacytidin[Title/Abstract]) OR azacytidine[Title/Abstract]) OR Decitabine[Title/Abstract]) OR “5-aza-2’-deoxycytidine”[Title/Abstract]) OR “5 aza 2’ desoxycytidine”[Title/Abstract]) OR “5 Deoxyazacytidine”[Title/Abstract]) OR “5-Deoxyazacytidine”[Title/Abstract]) OR Dacogen[Title/Abstract]) OR Ladakamycin[Title/Abstract]) OR mylosar[Title/Abstract]) OR nsc102816[Title/Abstract]) OR “nsc-102816”[Title/Abstract]) OR nsc127716[Title/Abstract]) OR “nsc 127716”[Title/Abstract]) OR vidaza[Title/Abstract])) OR “Decitabine”[Mesh]) OR “Azacitidine”[Mesh])

 © 2022. The Author(s).

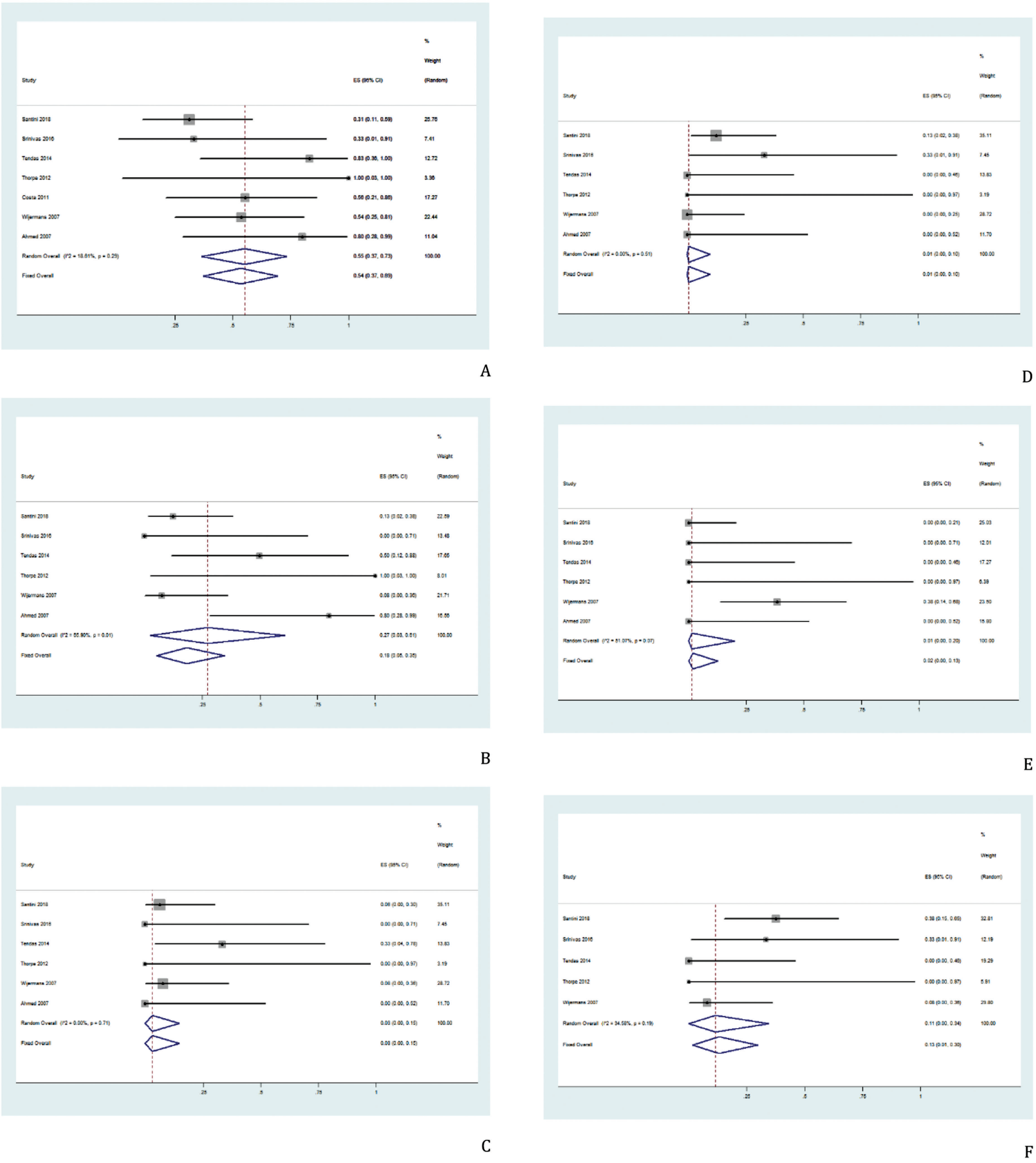

Supplementary Fig. S1 Pooled results of tumor response in the CMML-2 group. (**A**) Pooled results of ORR in the CMML-2 group was 54.0% (95% CI, 37.0–69.0%, $I^2 = 18.61\%$). (**B**) The pooled CR rate in the CMML-2 group was 27.0% (95% CI, 3.0–61.0%, $I^2 = 65.90\%$). (**C**) Pooled results of PR in the CMML-2 group was 3.0% (95% CI, 0.0–15.0%, $I^2 = 0.00\%$). (**D**) Pooled results of MR in the CMML-2 group was 1.0% (95% CI, 0.0–10.0%, $I^2 = 0.00\%$). (**E**) Pooled results of HI in the CMML-2 group was 1.0% (95% CI, 0.0–20.0%, $I^2 = 51.07\%$). (**F**) Pooled results of PD in the CMML-2 group was 13.0% (95% CI, 1.0–30.0%, $I^2 = 34.58\%$). CI, confidence interval; CMML, chronic myelomonocytic leukemia; CR, complete response; HI, hematologic improvement; ORR, objective response rate; MR, minor response; PD, disease progression; PR, partial response.

 © 2022. The Author(s).

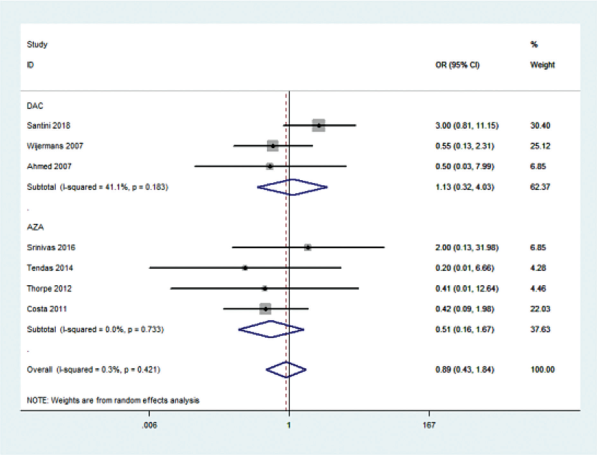

A

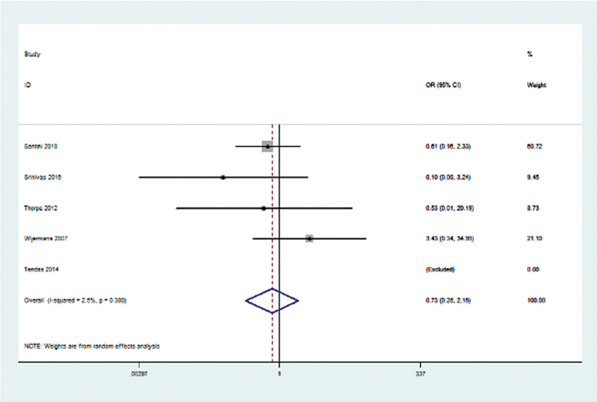

B

Supplementary Fig. S2 Difference in response rates between the CMML-1 and CMML-2 groups. (**A**) The difference in ORR between the CMML-1 and CMML-2 groups. (**B**) The difference in PD rate between the CMML-1 and CMML-2 groups. CMML, chronic myelomonocytic leukemia; ORR, objective response rate; PD, disease progression.

 © 2022. The Author(s).

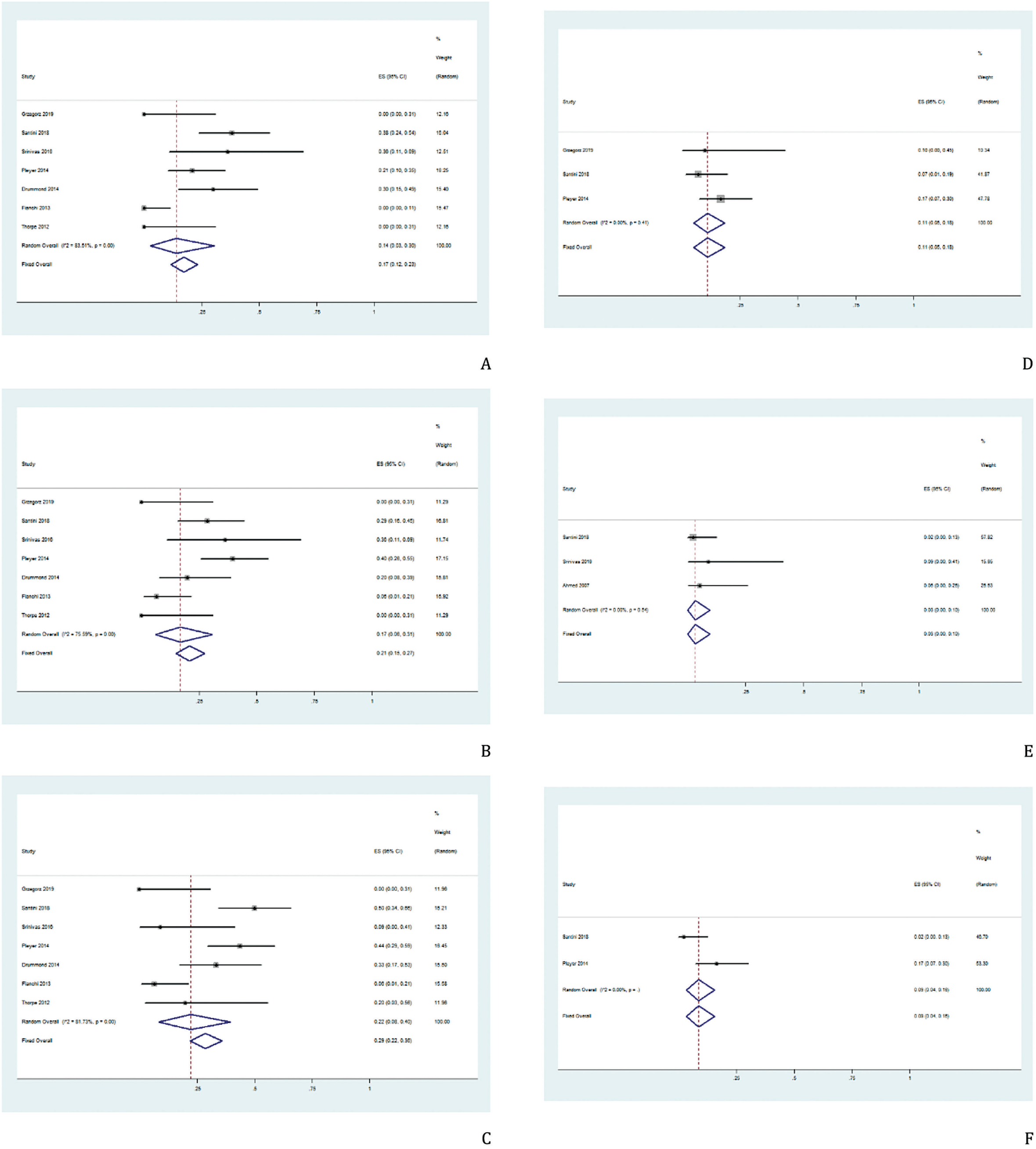

Supplementary Fig. S3 Hematological and nonhematological toxicity in total. (**A**)The pooled rate of grade 3/4 neutrophil toxicity was 14.0% (95% CI, 3.0–30.0%, $I^2 = 83.51\%$). (**B**)The rate of grade 3/4 anemia toxicity was 17.0% (95% CI, 6.0–31.0%, $I^2 = 75.59\%$). (**C**)The rate of grade 3/4 platelet toxicity was 22.0% (95% CI, 8.0–40.0%, $I^2 = 81.73\%$). (**D**) The pooled rate of infection was 11% (95% CI, 5–18%, $I^2 = 0.00\%$). (**E**) The pooled rate of bleeding was 3% (95% CI, 0–10%, $I^2 = 0.00\%$). (**F**)The pooled rate of cardiovascular diseases was 9% (95% CI, 4–16%, $I^2 = 0.00\%$). CI, confidence interval.

 © 2022. The Author(s).

Supplementary Table S1 Study characters

Study	County	Study type	Recruitment/ case review period	Follow-up time, months	Disease status	Intervention	Sample size	Age	Gender, male/female	End points	Criteria for response	Criteria for AEs
[20]	USA	Retrospective study	NR	55.5	CMML-0(50); CMML-1(35); CMML-2(35)	AZA(56); DAC(65)	56;65	68	76/45	ORR;CR;MR; PD;SD;CCyR	IWG	CTCAE
[22]	Poland	Retrospective study	2012-2016	9.4	CMML	AZA	10	67	7/3	ORR;CR;HI;PD; SD	IWG	CTCAE
[21]	Greece	Retrospective study	NR	25.9	CMML-1(58);CMML-2(30)	AZA	88	74.1	62/26	ORR;CR;PR;HI; PD;SD	IWG	CTCAE
[15]	Italy	phase II, multi-centre prospective trial	2010-2011	51.5	CMML-1(26); CMML-2(16)	DAC	42	71.5	30/12	ORR;CR;PR; MR;HI;PD;SD	IWG	CTCAE
[19]	USA	Retrospective study	2004-2015	17	CMML-0(40); CMML-1(58); CMML-2(52)	HMA	151	69	107/44	ORR;CR;PR; MR;HI;SD; CCyR	IWG	CTCAE
[18]	USA	Prospective non-random-ized phase 2 study	2010-2012	24	CMML-1(8); CMML-2(3)	AZA	11	69	6/5	ORR;CR;MR; PD;SD	IWG	CTCAE
[24]	Italy	Retrospective study	2010-2012	12.5	AML(2); CMML-1(2); CMML-2(5)	AZA	10	75	8/2	ORR;CR;PR;SD	IWG	CTCAE
[23]	Austria	Retrospective study	2009-2013	9.8	CMML-1(19); CMML-2(29)	AZA	48	71	29/19	ORR;CR;PR; MR;HI;SD	IWG	CTCAE
[16]	UK	A multi-centre phase 2 trial	2010.1-2010.8	12.8	CMML-1(21); CMML-2(8)	AZA	30	70	20/10	ORR;CR;MR; HI;PD;SD	IWG	CTCAE
[26]	Australia	Retrospective study	2008-2012	15.9	CMML-1(7); CMML-2(4)	AZA	11	65	NR	ORR;CR;PR; MR;HI;PD;SD	IWG	CTCAE
[25]	Italy	Retrospective study	2005-2011	NR	CMML-1(13); CMML-2(18)	AZA	31	69	23/8	ORR;CR;PR;HI; PD;SD	IWG	CTCAE
[27]	Australia	Retrospective study	2005-2010	NR	CMML	AZA	10	66	8/2	ORR;CR;HI;PD; SD	IWG	CTCAE
[28]	Austria	Retrospective study	1996-2008	NR	CMML-1(26); CMML-2(9)	AZA	36	70.5	29/7	ORR;CR;PR;HI; SD	IWG	CTCAE
[17]	3countries	One pivotal phase 3 trial and three phase 2 trials	NR	NR	CMML	DAC	31	71	23/8	ORR;CR;PR;HI; PD;SD;CCyR	IWG	CTCAE
[29]	USA	Retrospective study	2004-2005	NR	CMML	DAC	19	66	14/5	ORR;CR;HI	IWG	CTCAE

Note: CMML: chronic myelomonocytic leukemia. AML: acute myeloid leukemia. AZA: azacitidine. DAC: decitabine. ORR: objective response rate. CR: complete response. PR: partial response. MR: HI: PD: SD: OS: overall survival. IWG: CTCAE: Common Terminology Criteria for Adverse Events. SD: stable disease; CCyR: complete cytogenetic response.

 © 2022. The Author(s).

Supplementary Table S2 Quality assessment of included studies

A. MINORS index for included non-randomized studies.									
Study	I	II	III	IV	V	VI	VII	VIII	Total
[16]	2	1	2	2	2	2	2	0	13
[15]	2	1	2	2	2	2	2	0	13
[18]	2	1	2	2	2	2	2	0	13
[17]	2	0	2	2	2	2	2	0	12

B. JBI Critical Appraisal Checklist for Case Series for included retrospective studies											
Study	Q1	Q2	Q3	Q4	Q5	Q6	Q7	Q8	Q9	Q10	Overall appraisal
[20]	Y	Y	Y	Y	Y	Y	Y	Y	N	Y	Include
[22]	N	Y	Y	Y	Y	Y	Y	Y	N	Y	Include
[21]	N	Y	Y	Y	Y	Y	Y	Y	N	Y	Include
[19]	Y	Y	Y	Y	Y	Y	Y	Y	N	Y	Include
[24]	UN	Y	Y	Y	Y	Y	Y	Y	N	UN	Include
[23]	Y	Y	Y	Y	Y	Y	Y	Y	N	Y	Include
[26]	Y	Y	Y	Y	Y	Y	Y	Y	N	UN	Include
[25]	Y	Y	Y	Y	Y	Y	Y	Y	N	UN	Include
[27]	N	Y	Y	Y	Y	Y	Y	Y	N	UN	Include
[28]	Y	Y	Y	Y	N	Y	Y	Y	N	Y	Include
[29]	Y	Y	Y	Y	N	Y	Y	Y	N	Y	Include

Note: numbers I-VIII in heading signified: I, a clearly stated aim; II, inclusion of consecutive patients; III, prospective collection of data; IV, endpoints appropriate to the aim of the study; V, unbiased assessment of the study endpoint; VI, follow-up period appropriate to the aim of the study; VII, loss of follow up less than 5%; VIII, prospective calculation of the study size.
Note: numbers Q1-Q10 in heading signified: Q1, were there clear criteria for inclusion in the case series? Q2, was the condition measured in a standard, reliable way for all participants included in the case series? Q3, were valid methods used for identification of the condition for all participants included in the case series? Q4, did the case series have consecutive inclusion of participants? Q5, did the case series have complete inclusion of participants? Q6, was there clear reporting of the demographics of the participants in the study Q7, was there clear reporting of clinical information of the participants? Q8, were the outcomes or follow up results of cases clearly reported? Q9, was there clear reporting of the presenting site(s)/clinic(s) demographic information? Q10, was statistical analysis appropriate?

 © 2022. The Author(s).
